# Evaluating Research for Development: Innovation to Navigate Complexity

**DOI:** 10.1057/s41287-023-00577-x

**Published:** 2023-03-01

**Authors:** Marina Apgar, Mieke Snijder, Grace Lyn Higdon, Sylvia Szabo

**Affiliations:** 1 Institute of Development Studies, Brighton, UK; 2 Monitoring, Evaluation and Learning Advisor, Independent, Eastbourne, UK; 3 Department of Social Welfare Counseling, Dongguk University, Seoul, South Korea

**Keywords:** Research for Development, Complexity, Innovation, Causal Pathways, Learning

## Abstract

Large publicly funded programmes of research continue to receive increased investment as interventions aiming to produce impact for the world’s poorest and most marginalized populations. At this intersection of research and development, research is expected to contribute to complex processes of societal change. Embracing a co-produced view of impact as emerging along uncertain causal pathways often without predefined outcomes calls for innovation in the use of complexity-aware approaches to evaluation. The papers in this special issue present rich experiences of authors working across sectors and geographies, employing methodological innovation and navigating power as they reconcile tensions. They illustrate the challenges with (i) evaluating performance to meet accountability demands while fostering learning for adaptation; (ii) evaluating prospective theories of change while capturing emergent change; (iii) evaluating internal relational dimensions while measuring external development outcomes; (iv) evaluating across scales: from measuring local level end impact to understanding contributions to systems level change. Taken as a whole, the issue illustrates how the research for development evaluation field is maturing through the experiences of a growing and diverse group of researchers and evaluators as they shift from using narrow accountability instruments to appreciating emergent causal pathways within research for development.

## Résumé

Les grands programmes de recherche financés par des fonds publics continuent de recevoir des investissements accrus en tant qu'interventions visant à produire un impact pour les populations les plus pauvres et les plus marginalisées dans le monde. À cette intersection entre la recherche et le développement, la recherche devrait contribuer aux processus complexes de changement sociétal. Pour adopter une vision coconstruite de l'impact comme phénomène émergeant au fil de liens de causalité incertains, bien souvent sans résultats prédéfinis, il faut innover en utilisant des approches d'évaluation sensibles à la complexité. Les articles de ce numéro spécial présentent de riches expériences d'auteurs travaillant dans différents secteurs et zones géographiques, employant l'innovation méthodologique et le pouvoir de navigation tout en réconciliant les tensions. Ils illustrent les défis lorsqu’il s’agit (i) d'évaluer des performances pour répondre aux exigences de redevabilité tout en favorisant l'apprentissage pour l'adaptation; (ii) d’évaluer les théories prospectives du changement tout en saisissant le changement émergent; (iii) d’évaluer les dimensions relationnelles internes tout en mesurant les résultats de développement externes; (iv) d’évaluer à différentes échelles: de la mesure de l'impact final au niveau local à la compréhension des contributions au changement au niveau des systèmes. Pris dans son ensemble, ce numéro illustre la façon dont l'évaluation de la recherche pour le développement mûrit à travers les expériences d'un groupe toujours plus important et divers de chercheurs et d'évaluateurs qui abandonnent des outils de redevabilité étriqués afin d’apprécier les liens de causalité émergents au sein de la recherche pour le développement.

## The R4D Evaluation Challenge and Opportunity

The aim of research for development (R4D) is to use research as a vehicle to address critical development concerns, in order to improve the lives and livelihoods of disadvantaged communities across the world. R4D programmes are neither purely academic research nor are they discrete development interventions. As hybrid research endeavors they are expected to contribute to complex processes of societal change, and achievement of development outcomes in particular. Many large R4D programmes that are funded through international development (aid) budgets or philanthropic institutions are vast in size, scope, and ambition. Some have a long history, such as the CGIAR system of agricultural research which has received $60 billion in investment over more than 40 years.^1^ More recent forms of ‘challenge driven’ research include research aimed at the UN Sustainable Development Goals (SDGs) (Borrás 2019), the €80 billion^2^ European Union Horizon 2020 research and development programme (Mazzucato et al. 2018), the €10 billion^3^ WWWforEurope project (Aiginger and Schratzenstaller 2016) and the £1.5 billion Global Challenges Research Fund (GCRF) funded by the United Kingdom government’s Department of Business, Energy and Industrial Strategy (BEIS) (Barr et al. 2019) which includes 3000 awards within a highly diverse portfolio that spans across all SDGs and lower-middle income countries (LMICs). The inter- and transdisciplinary research funded under these windows is framed around addressing ‘societal grand challenges’ requiring work across scales and sectors. At their core, R4D programmes are funded to undertake research as an intervention that produces direct, real-world impacts for the world's poorest and most marginalized populations.

In today’s context of performance-based research funding, demand for evaluation of the impact of academic research has increased (Zacharewicz et al. 2019; Bornmann 2013) leading to ever more sophisticated metrics for assessing the excellence of research (Pinar and Horne 2022). The focus of these assessments, however, is premised on a linear pathway starting from new knowledge produced by excellent academic research, communicated through engagement activities and subsequently leading to changes in policy (see Georgalakis and Rose 2019 for broader debates on understanding how research leads to policy impact). Evaluation of agricultural research for development programmes have similarly been driven largely by assumptions of linear technology impact pathways that fail to engage with the complexity of outcomes that emerge through social interactions of multiple actors in agricultural systems (Belcher and Hughes 2021). This linear and overly simplified view of research impact pathways continues to dominate even as research is expected to engage with broader processes of change.

**Fig. 1:**
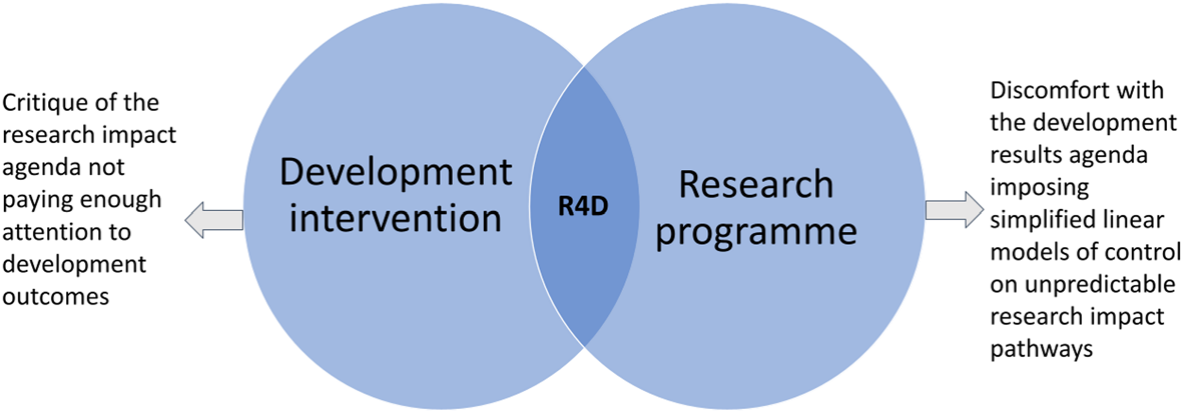
R4D programmes sit at the intersection of research and development

R4D funded via aid budgets experiences even higher levels of scrutiny on effectiveness given the prevalence of the development ‘results agenda’ (Eyben et al. 2015). As shown in Fig. 1 R4D programmes sit at the intersection of the research and development sectors creating pressures from both the research and the development impact agendas. A key task for evaluators is to reconcile research excellence with development effectiveness. On the one hand, part of the academic research community are uncomfortable with the imposition of a linear development evaluation orientation that doesn’t fully appreciate the unpredictability of research impact pathways^4^ (Eyben et al. 2015). On the other hand, part of the development community, which is used to a results-based-management framing (e.g. Hatton and Schroeder 2007) finds the research excellence evaluation agenda not sufficiently focused on the harder to measure downstream development outcomes that move beyond policy change to real-world impact on people's lives and livelihoods (Peterson, 10.1057/s41287-022-00565-7).

Decades of R4D theory and practice has highlighted the complexity of the impact pathways of these hybrid programs (Horton an Mackay 2003; Thornton et al. 2017). They contain a multitude of actors that are engaged throughout the knowledge production process, moving away from a linear view of the discovery-to-application pipeline. Moving from simpler views of using the products of excellent research in knowledge exchange, to a co-produced view of impact along uncertain pathways, requires innovation in the way programmes are designed, operationalised, and consequently, evaluated (Blundo-Canto et al. 2017; Jacobi et al. 2020; Maru et al. 2018; Temple et al. 2018). Further, the intention around inclusion of marginalized voices and perspectives in how outcomes are achieved, requires systems approaches that open up opportunities for alternative pathways to emerge (Leach et al. 2007) with research sometimes acting as a disruptive force through which development is achieved (Ely et al. 2020).

Monitoring, Evaluation and Learning (MEL) systems that are fit for purpose for R4D programming must work with the complexity that arises from a large number of diverse partners working together in many and often integrated work streams, on problems in different contexts and on research that may have no predefined outcomes. This poses challenges to traditional evaluation designs that use a before and after (baseline-endline) logic, or seek counterfactual evidence of effectiveness, and requires acknowledgement of evaluation as embedded and enmeshed in complex social and political dynamics. The increased interest and funding for R4D has created an exciting opportunity to learn from experimentation with new evaluation designs and practices that are contributing to middle range theories (see Cartwright 2020 for a full explanation) of how R4D programmes work as well as evaluation theory and practice.

This special issue originated from a series of intentional learning exchanges by researchers and evaluators engaged in evaluation of programmes funded under the UKRI GCRF. The resulting special issue covers papers that shed light on different methods, approaches and areas of evaluation in R4D, including evaluation of specific funder portfolios, relational aspects of R4D, the link between R4D and SDGs and the use of theory of change and learning approaches. It is the first consolidated examination of R4D evaluation theory and practice, responding to the expansion of this field of funding and practice in the UK and beyond. In this introductory editorial we first discuss the R4D evaluation landscape as situated within a broader shift towards being more complexity-aware and better able to navigate uncertain impact pathways. We then describe four areas of tension that are experienced as challenges within complexity-aware evaluation practice and introduce the papers in this special issue by showing how they address each. Finally, we share our reflections on the future of R4D evaluation as editors of this special issue.

## The Shifting Landscape of Complexity-Aware Evaluation

The implications of complexity for examining if and how interventions, research among them, lead to societal changes (outcomes), are increasingly recognised in evaluation theory and practice across a number of fields (Walton 2016). In the international development sector, embracing the SDGs as an overarching framework generated momentum around rethinking impact evaluation (e.g. Befani et al. 2014) to broaden beyond what until then had been narrow views of experimental designs as the ‘gold standard’. The so-called Stern review (Stern et al. 2012) commissioned by the UK Department for International Development (now the Foreign and Commonwealth Development Office) was pivotal in highlighting the need to nuance our understanding of methods through engaging with the underpinning frameworks used for making a causal claim. Subsequently, others have built on this foundation (see Gates and Dyson 2017; Jenal and Liesner 2017; Masset et al. 2021) to illustrate that evaluators working in conditions of complexity can choose from a range of approaches and methods, underpinned by distinct causal frameworks.^5^ Table 1 illustrates the diversity in available designs.

**Table 1:** Evaluation approaches and methods and their causal frameworks

Approach	Examples of methods	Underpinning causal framework
Theory-based approaches	∙ Contribution analysis ∙ Process tracing ∙ Realist evaluation ∙ General elimination methodology ∙ Qualitative impact assessment protocol ∙ Innovation history	In-depth theoretical analysis of causal processes or mechanisms in context. Many of these methods are grounded in a generative causal framework, though configurational frameworks are also relevant to some
Participatory approaches	∙ Most significant change ∙ Outcome harvesting ∙ Collaborative outcomes reporting ∙ Collaborative yarning	Engagement of change agents (programme participants) in defining the scope of change to be explored and in analysis of causal claims. Many of these methods are grounded in a generative causal framework
Case-based approaches	∙ Within-case ∙ Across-case	Analysis of causal processes within a case or across multiple cases based largely on a configurational causal framework though generative frameworks are also relevant (especially when combined with theory-based approaches)

Adapted from Jenal and Liesner (2017), Lynn et al. (2021, Lynn and Apgar (forthcoming)

In the social change sectors, including philanthropy, there is a noticeable turn to systems interventions or systems change strategies, leading to further theoretical and practice developments in the nascent field of evaluating systems change (Gates 2017; Hargreaves and Podems 2012; Lynn et al. 2021; Walton 2016). Related is the move towards innovation oriented and complexity informed programming (e.g. Burns and Worsley 2015; Jones 2011; Ramalingam 2013) calling for appropriate evaluation designs. We see growing demand and use of a family of evaluation approaches that are ‘complexity-aware’ including the well-known developmental evaluation (Patton 2010) and associated principles-focused evaluation (Patton 2017). They emphasize learning about how outcomes emerge along unpredictable impact pathways, with the intention of feeding learning back into implementation.

In the context of systemic, learning oriented and adaptive programming, there is no obvious evaluation design, and evaluators must work with programmers to choose and tailor appropriate designs from the variety of options available to them. Established guidance suggests methodological choice should be appropriate for the evaluation questions and the attributes of the intervention (HM Treasury; Befani 2020). And a number of typologies (e.g. Masset et al. 2021) and checklists (e.g. Bamberger et al. 2016) have been developed that acknowledge complexity should inform methodological choice. More recently, the term ‘bricolage’ is being used to guide evaluators in not only choosing and mixing methods, but to recombine different parts of methods (Aston and Apgar 2022; Hargreaves 2021) to support rigour in making causal claims amid complexity.

Another way in which evaluators are responding to complexity is the move towards greater use of theory-based approaches which start with an articulated theory of how an intervention is thought to achieve impact (Rogers and Weiss 2007; Weiss 1997). A wide range of methods (see Table 1) fit within this family and have particular ways of developing theory, causal assumptions and testing or refining them through evaluation research. A core contribution of these approaches, and in particular realist evaluation (Pawson and Tilley 1997) and process tracing (Beach 2017; Stachowiak et al. 2020) is acknowledging contextual conditions as part of the causal relationships under investigation. Underpinned by configurational and generative causal frameworks, they are well suited to answer evaluation questions about not just what, but how, in what conditions and for whom are outcomes and impact achieved. Further, these approaches can support learning through iterative use of theory of change (ToC), as illustrated by evolving approaches to contribution analysis (Apgar et al. 2020; Ton et al. 2019).

A common theme across these learning-oriented approaches to evaluating large and complex programmes is to appreciate evaluation design as an iterative process rather than a single decision point at the outset. Evaluators working in conditions of complexity must evolve their designs as outcomes emerge and assumptions about causal links are clarified, and as learning agendas are reshaped along the way through collaboration with stakeholders. This has implications for the capacities required as evaluators must shift their role from being external technical experts to embedded facilitators (Barnett and Eager 2021). The move away from evaluation as simply a technical endeavor, to embracing the politics within the evaluation process and how it informs decision making (Eyben et al. 2015; Polonenko 2018) is reopening long standing debates about whose knowledge counts in evaluation (see Estrella et al. 2000). Related calls for greater equity-orientation in evaluation (Forestieri 2020; Gates et al. 2022; Hall 2020) are focusing attention on power ‘in’ and ‘of’ evaluation (Hanberger 2022). These trends are creating new opportunities for re-centering the role of the evaluator as a knowledge broker engaging with commissioners, programmers and change actors within systems.

Evaluating R4D programmes sits at the intersection of research and development, and takes place within an established yet still evolving landscape of learning oriented and complexity-aware evaluation practice. Exploring the uncertain impact pathways of R4D requires navigating difficult methodological choices and managing across distinct, at times contested fields of practice and thinking enmeshed in politics and power. The papers in this issue help us to understand what these tensions look like through experiences of diverse teams working at different scales and across contexts. In the following section we introduce the papers through four interconnected tensions, grounding each in the literature and highlighting how papers in this issue contribute to these areas of contestation, debate and praxis within the field of R4D evaluation.

## Navigating Tensions in R4D Evaluation

We organize our introduction to the papers in this special issue by describing four areas of tension that are experienced as challenges within complexity-aware evaluation practice. The four areas span the realms of decision making span the realms of decision making on focus and purpose and related decisions on appropriate methodological choices, all the way through to the way in which evaluation findings are used and the strength of causal claims are assessed. While the four areas of tensions are in practice interconnected, for ease of presentation we explore each separately and introduce the papers featured in this issue as they contribute to R4D evaluation theory and practice.

### Evaluating Performance to Meet Accountability Demands While Fostering Learning for Adaptation

The tension between learning and accountability is well recognised in the context of development evaluation (Estrella et al. 2000; Guijt 2010; Guijt and Roche 2014) and links to broader debates around the politics of evidence and the narrow framing of the ‘results agenda’ in international development (Eyben et al. 2015). This tension is particularly relevant in how the effectiveness of newly developed hybrid challenge-driven research programmes, which are implemented by academic institutions and funded through aid budgets, is understood and consequently, how programmes are evaluated. A strict performance management and narrow accountability focus—be that on research excellence or development outcomes—can inhibit open and honest sharing of successes and failures and shut down the space required for learning to drive the generation of innovation—one of the aims of R4D. These poor conditions are brought into relief through technical challenges, such as traditionally used methods for performance monitoring and management, which rely on linear and predefined models of performance, which focus solely on academic output, combined with political challenges inherent within different stakeholders’ epistemological beliefs, particular agendas, and influence within a given program that shape or winnow the type of learning that can be generated (Aston et al. 2021).

For years—and to varying degrees of success—communities of practice seeking to understand complex, learning-oriented initiatives have included stakeholders interested in measuring policy and advocacy, thinking and working politically, and adaptive management. Practitioners have been debating approaches and practicing methods to navigate these tensions. Activities embedded within complexity-aware evaluation designs (see Table 1) include structured reflective moments conducted in intervals that feed into decisions made during implementation, thus providing documentation and justification for ‘real-time’ adjustments which address accountability requirements while simultaneously providing opportunities for program stakeholders to take stock and collectively learn. Other activities include capturing observed progress toward outcome level changes during implementation through participatory data collection and synthesis processes which provide evidence to understand program performance while also allowing program stakeholders to appreciate what and how outcomes are emerging (Laws and Marquette 2018; Pasanen and Barnett n.d.; Reisman et al. 2007).

From decades of experience we know that the space for learning requires more than simply having the right tools. The political conditions must also be favorable: participatory and qualitative evidence have to be considered valid, reporting failure and acknowledgement of uncertain predictions must be considered acceptable, and course corrections-accompanied by diverting funds-must be allowed. In recent years, while uptake of more learning-oriented approaches has grown, the efficacy of those approaches can be drastically limited by the program’s funding environment if the environment remains premised on linear programme modalities with predicted milestones (McCullough et al. 2017).

Few tools illustrate this juxtaposition better than the recent uptake of the use of ToC over the past decade (Vogel 2012). For some, the use of ToC represents a mechanism of resistance to strict indicator-based results frameworks, allowing for more collaborative, nuanced and non-linear predictive program modeling that does not pin programs down to milestones (Apgar et al. 2022). In others’ experience, ToC has been co-opted and is yet another version of a results matrix (e.g. logframe 2.0), especially in cases where funders require performance predictions to be tied to the ToC at the outset of the program and have low appetite for adaptation of the ToC during implementation.

Chapman et al. (this issue) explore the role of ToC in navigating the tensions between learning and accountability in R4D evaluation through examination of the use of visual ToCs by MEL practitioners and evaluators working on ten large R4D programmes funded under the GCRF. The authors conclude across all experiences, that due to the performance management requirements- including output-focused results reporting required by the funder—most of the 10 programmes opted to simplify their visualizations and overall use of ToCs. Although some set out to use ToCs as strategic learning tools, due to capacity issues, resource constraints, and the political operating environment of these R4D programmes, simplified ToCs were found to be most useful to establish standardized language across multi-disciplinary teams, depict ‘big picture’ programmatic goals, and inform logical framework design—not as learning tools. The paper highlights how the accountability environment of R4D programmes may challenge the intention to use ToC as a learning tool.

Apgar et al. (10.1057/s41287-023-00576-y) explore the use of Social Network Analysis (SNA) in three large scale R4D programmes as a tool for learning about how the programme structures evolved through time (linked to performance and programme assumptions in ToC) while also using the learning to intentionally weave the networks in desirable directions (adaptation). Through the three case experiences they reveal tensions between these two purposes especially in how SNA findings are interpreted and by whom, and conclude that “navigating the challenges of interpretation and ethical dilemmas requires careful consideration as well as an enabling institutional and political environment for use of SNA to support learning.” (Apgar et al. 10.1057/s41287-023-00576-y pxy). They recommend embedding the interpretation of SNA findings into participatory sense making moments within the broader adaptive management designs of R4D programmes so as to hold open sufficient space for learning to be actioned.

Embracing complexity and moving away from standardized and predefined accountability metrics requires R4D evaluation stakeholders to decide first what it is they value, in order to build an appropriate approach to learn as outcomes emerge. Peterson (10.1057/s41287-022-00565-7) shares an alternative to ‘Value for Money’, one of the main instruments of accountability, particularly prevalent in the current constrained UK government spending environment. Peterson identifies the need to bridge between standard econometric evaluation designs and complexity-aware methodologies to build an approach that is fit for purpose. She presents a collaboratively developed rubric-based approach to reviewing R4D projects, couched in a constructivist paradigm that allows for valuing both the process and the outcomes of research, and reflects on its application in two large R4D portfolios. The paper illustrates how accountability and learning can be brought together through methodological innovation.

### Evaluating Prospective Theories of Change While Capturing and Exploring Emergent Change

Building on the use of ToC as a learning and management tool, is the now common practice of using ToC to inform theory-based approaches to impact evaluation (see Table 1). Yet being theory based does not necessarily lead to being complexity- aware. Indeed, there is often a tension experienced between prospective and retrospective use of ToC, with a separation of monitoring tools to track predefined indicators within a ToC that looks forward and evaluation aiming to understand results looking backwards. As Jenal and Liesner (2017) note, a key criticism of prospective approaches to ToC in evaluating systems change is that they do not capture unexpected changes. Doing deep causal thinking at the outset of a programme and detailing causal theories of change is helpful to guide evaluation research and zoom into specific causal links within a ToC. But, R4D impact pathways are complex and unpredictable, leading often to unexpected effects which prospective use of ToC may miss if applied in an overly linear way. In particular, the visualization of ToC bears the risk of oversimplification and the unintended effect of pushing a linear causal logic that crowds out any space for emergence (Davies 2018; Wilkinson et al. 2021).

Chapman et al. (this issue) reflect on how to balance funder requirements for a simplified ToC with one that embraces the complexity of R4D and leaves space for emergence. They share two strategies to navigate this tension. Firstly, some of the programmes used nested ToCs, with a linear overarching ToC that was then broken down into specific ToCs detailing how a specific component of the R4D programme was contributing to specific outcomes and impacts or how outcomes and impacts were going to be achieved in a specific geographical area of the programme. These nested ToC were more adaptable and manageable than the large programme level ToC and gave the programme space to embrace more uncertainty and emergence in the evaluation designs. Secondly, one programme used visualized impact pathways combining system mapping with ToC and participatory approaches that allowed for iterative revisions, non-linearity and feedback loops. While a resource intensive approach, it illustrates the need to innovate with ToC when working with large complex projects to keep the space open for the exploratory nature of R4D.

An additional challenge with prospective approaches to ToC is that they can suffer from overconfidence in projecting a single anticipated future that hides other contributory factors. This is a form of confirmation bias often critiqued when evaluating complex processes of change. The Impact Weaving method introduced by Blundo-Canto et al. (10.1057/s41287-022-00566-6) is a way to embrace complexity in ToC by updating researcher-generated impact pathways of agricultural innovations with other stakeholders’ knowledge. This method plays with prospective and retrospective aspects of the impact pathways under investigation by combining past knowledge from researchers and other stakeholders, with knowledge of the current situation and context from stakeholders who are or will be using the innovations, with future knowledge through visioning and participatory scenario building. This transdisciplinary method creates contextually relevant impact pathways that provide a more systemic, triangulated and grounded vision of how change actually happens.

Often combined with prospective approaches to ToC in theory-based evaluation are goal independent evaluation methods such as Outcome Harvesting (Wilson-Grau 2018, p.) and Most Significant Change (Davies and Dart 2005) that capture emergent change by identifying outcomes after they have been produced, and tracing contribution back to the programme under evaluation. These are forms of causes-of-effects analysis often found in case-based methods (Goertz and Mahoney 2012). In this issue, a new method that aims to combine prospective and retrospective approaches in the context of policy evaluations is introduced by Douthwaite et al. (10.1057/s41287-022-00569-3) called outcome trajectory evaluation (OTE). The aim of OTE is to cover all factors that are hypothesized to be influenced by policy outcomes, not just the ones that are targeted by the policy. In OTE outcomes are “understood to emerge in complex adaptive systems, through the interaction of actors, their strategies and decision-making, institutions, artifacts (i.e., technology) and knowledge” (Douthwaite et al. 10.1057/s41287-022-00569-3). This view of outcomes embraces emergence and systems change at its core by taking a long-term view of outcomes, rather than seeing outcomes as a single episode of change. This offers a more balanced view of the contribution of any specific intervention within context.

### Evaluating Internal Relational Dimensions While Measuring External Development Outcomes

R4D programmes, through their hybrid nature, require collaboration across diverse actors and sectors—from research institutions and researchers of different disciplines to development agencies, government departments and practitioners—to focus together on addressing grand societal challenges (or SDGs) often working across contexts. These large inter- or transdisciplinary collaborations create relational spaces through which impact is enabled downstream, often taking a long time to materialize. This has been referred to as the ‘productive interactions’ space between research and other societal actors and has been informing the evaluation of European research systems (Muhonen et al. 2020).

In these large R4D collaborations, it is such productive interactions that create the conditions for outcomes to emerge, rather than achievements of individual actors leading to discrete outcomes (Hargreaves 2021; Walton 2016). The relational components are inherently unpredictable, requiring, as noted already, an emphasis on learning real time as relationships evolve and opportunities for impact become clearer. Building on the tension already discussed around accountability versus learning, a focus solely on measuring ‘results’ downstream misses the opportunity to understand the conditions through which they emerge. Within complexity aware evaluation approaches, evaluation questions of how and why outcomes are emerging are well placed to inquire into the relational as mechanisms for achieving impact.

Specific approaches to exploring these relational dynamics are gaining ground, for example, appreciating the processes of co-production in interdisciplinary research programmes (de Sandes-Guimarães et al. 2022). Processes of co-production are premised on productive and equitable collaborations across actors—in other words the internal ‘ways of working’. Snijder et al. (10.1057/s41287-023-00578-w) focus specifically on evaluation and learning of equitable partnerships through a comparison across five large scale R4D programmes. Across the five cases they illustrate how decolonial, feminist and participatory approaches were used to address hard to shift power asymmetries related to: funding flows from the so-called ‘global north’ to lower and middle income countries; hierarchies between senior researchers and early career researchers as well as across disciplines and genders. The authors argue that participatory approaches embedded in programme MEL allow internal power dynamics to be revealed and acted upon in support of adaptive management, and they propose a framework that distills key principles for evaluating equitable partnerships in R4D programmes.

Looking across the whole GCRF in evaluating the early phases of implementation, Vogel and Barnett (10.1057/s41287-023-00579-9) share emerging evidence on how conditions for R4D impact are built, shining a light on the processes of set up and implementation as mechanisms for impact down the road. They identify four building blocks as “elements and processes that projects need to build into their research to position it for impact.” (Vogel and Barnett, 10.1057/s41287-023-00579-9, p. X): (1) Scoping of development issues with stakeholders on the ground for relevance; (2) Fair and equitable partnerships between partners in the so-called Global North and lower and middle income countries, including non-academic partners, integrating mutual capacity building; (3) Gender, social inclusion and poverty prioritized in policies and implementation, and (4) Stakeholder engagement in lower and middle income countries to support positioning research for use. In using the GCRF as a case of R4D they echo Snijder et al. (10.1057/s41287-023-00578-w) by highlighting the efforts and success of the signature GCRF Hubs in setting up mechanisms to ensure fair representation of partners within internal governance and decision-making as well as evaluation of equity in these partnerships.

This focus on the internal dynamics of large multidisciplinary research programmes is leading to use of novel methods in evaluating research teams and how they work, such as SNA (e.g. (Higgins and Smith 2022). Apgar et al. (10.1057/s41287-023-00576-y) zoom into the relational structures of R4D programmes through evaluating them as network building initiatives. The SNA method, employed within MEL systems designed to inform adaptive management, revealed how the network structures matched the expectations of a centralized setup of the programmes with central coordination teams in the Global North. It also brought to light surprising evolutions in the structures that enabled questioning of underlying assumptions, for example, around the gendered dynamics of collaboration. The authors suggest that paying more attention to the relational at the outset, through developing ‘contextualized theories of collaboration’ would enhance the use of SNA in evaluation through guiding studies in more purposeful ways.

### Evaluating Across Scales: From Measuring Local Level End Impact to Understanding Contributions to Systems Level Change

Another lens through which tensions can be felt and analyzed in R4D programmes relates to the scales at which impact is theorized to emerge and consequently evaluated. Like complex systems, R4D programmes include multiple levels of ‘nested’ interventions within them. To inform future investments at the fund level, portfolios of projects are evaluated to assess what has been achieved overall and how large scale interventions work, and within a fund, individual programmes or projects of varying sizes focused on particular challenges in contextualized ways and are evaluated to assess their outcomes and impact and to learn how to improve R4D interventions. R4D funds are increasingly framed around the SDGs suggesting ‘global level’ impact as the end goal at the portfolio level. Yet these global goals need to be materialized in concrete changes in people's lives and livelihoods—such as poverty reduction—which point to measuring real change in specific locations linked to individual interventions. The levels are interconnected, creating unique challenges for evaluation design.

In the context of the UK, the Newton Fund and the GCRF evaluations offered opportunity to innovate, connecting across project and fund level evaluations. Two papers in this issue share learning from these evaluations as informative cases of large R4D portfolios. Peterson (10.1057/s41287-022-00565-7) details the steps used to build a methodology for portfolio level assessment of Value for Money based on multiple-case studies of individual projects. The cases provide in-depth details of R4D impact, which enables comparison across cases while being attentive to the uniqueness and contextuality of each case. Rubrics were developed with all stakeholders to agree what criteria to value, a method that allows tailoring to the specific needs of R4D programmes with criteria such as ‘equitable partnerships’, and ‘likelihood of fund level impact’ to allow connecting across the project and fund levels. Vogel and Barnett (10.1057/s41287-023-00579-9) share emerging evidence from the GCRF fund level evaluation which is still ongoing. The evaluation team in the early phases had to navigate the diversity produced by a highly devolved structure of the fund which is delivered through 17 partners through existing research and University systems. The resulting 3000 awards make up a highly diverse portfolio that spans across all SDGs and LMICs. The ‘building blocks’ for impact they identify are the result of synthesis across many case studies through their modular evaluation design and provide insights on core mechanisms for impact of R4D programmes.

The evaluation community has been grappling with the SDG agenda and pushing towards evaluation being at the service of transformative change in systems (Aronsson and Hassnain 2019)). The challenge of scale is evident in the complexity of setting SDG targets and indicators. Some experts have suggested a two-track solution to measuring SDGs would be most efficient, allowing for both ‘goal level’ measures and lower level ‘technical indicators’ that could relate to interventions more directly (Davis et al. 2015). Gonzales et al. (10.1057/s41287-022-00573-7) engage with the challenge of measuring contribution of an R4D project on trade and environment. They propose a methodology for mapping specific contributions of the project through its planned outcomes areas (named ‘big wins’) to the SDG targets. This detailed method links to the evaluation design, and is able to provide a clear line of sight between outputs of every work package within the project to system level measures of impact.

## What Next for R4D Evaluation?

We have described R4D programmes as sitting at the intersection of research and development (see Fig. 1) and the papers in this issue present rich experiences of a community of evaluation practitioners and researchers that are innovating methodologically to navigate and reconcile the tensions that arise from this hybrid reality. As Vogel and Barnett (10.1057/s41287-023-00579-9) note, the GCRF evaluation is framing this hybrid reality through a unifying construct of ‘development excellence’ as the main goal of R4D programmes. We understand R4D evaluation as an evolving field and this issue is the result of evaluators, programme managers and researchers exploring the practical implications of navigating these tensions, often with little formal guidance and facing many hurdles along the way. This growing community of R4D evaluation researchers is represented in this special issue by over 40 co-authors from over 10 countries in Europe and middle- and low-income countries, including a significant number of early career researchers. Through the papers, the voices of this community come to life, acknowledging the embeddedness of R4D evaluation, and the reflexivity required of diverse evaluation teams.

Taken together, the papers in this issue provide insights on how complexity-aware and ‘bricolaged’ evaluation designs are implemented in the context of R4D programmes. In the process of developing this special issue, we, the editorial team, and all co-authors engaged in the GCRF programmes experienced a major funding crisis that served to sharpen our empirical understanding of the challenge of engaging in the contested spaces we have described here as areas of tension. In November 2020, in response to the COVID-19 pandemic, the UK Aid budget was significantly reduced, and as a consequence the funding of the 12 signature GCRF Hubs was cut by up to 70% (UKRI 2021; Nwako et al. 2023). For a period, the implementation teams faced high levels of uncertainty about future funding and the viability of the evaluation research. This led to a sudden shift away from the learning orientation that had informed evaluation designs and practice. Additional reporting requirements were placed upon all programmes, with new hoops to be jumped through that forced a reorientation on proving what had been achieved and away from exploring the ways in which impact opportunities were emerging along unpredictable pathways. The space for deepening learning around the relational and internal dynamics was curtailed and the reduced budgets meant in some cases MEL systems and expertise was no longer a priority.

This recent GCRF experience highlights the double-edged sword of evaluation as a performance management tool and a research and learning tool. And it is not entirely unique. Indeed, evaluators working within the CGIAR system have at times experienced similar shifts in the use of evaluation in response to reduced funding. Douthwaite et al. (2017) describe one such experience which led to a shift away from systems-oriented research programmes, arguing that underpinning the shift was a different way of valuing R4D programmes, based on a narrow view of causal claims requiring counterfactual designs. As Peterson et al. (10.1057/s41287-022-00565-7) show, there is still room to deepen the shift away from the underlying positivist leaning in the accountability instruments used to assess the value of research to see greater uptake of methods that are more fit for purpose.

Despite, or perhaps because of, the challenges that remain, the papers in this issue illustrate that reconciling tensions in R4D evaluation is possible, offering methodological innovations that show in practice that the trend towards broadening evaluation designs to embrace complexity is gaining momentum. Related calls in the sustainability field for greater funding flexibility to stay the course, to give time and space for the impacts of systemic and transdisciplinary research to materialize downstream (e.g. Ely 2021; Benedum et al. 2022) and testing of funder developed approaches to evaluating research such as the IDRC Research Quality + tool (McLean et al. 2022; Lebel and McLean 2018) provides further grounds for optimism. The field of R4D evaluation will continue to mature and this issue illustrates that diverse experiences and learning across R4D stakeholders, including researchers, research managers, evaluators and funders is contributing to its coming of age.

## Footnotes

1 https://www.cgiar.org/annual-report/performance-report-2020/assessing-cgiars-return-on-investment/.

2 https://www.eeas.europa.eu/eeas/horizon-2020_en#:~:text=Horizon%202020%20is%20the%20EU's,(roughly%20JPY%2010%20trillion).

3 https://cordis.europa.eu/project/id/290647.

4 See the LSE impact blog for a longstasnding conversation on how to measure academic impact: https://blogs.lse.ac.uk/impactofsocialsciences/the-handbook/chapter-6-is-there-an-impacts-gap-from-academic-work-to-external-impacts-and-perhaps-also-to-consequences-how-might-it-have-arisen-how-might-it-be-reduced/.

5 See Stern et al. (2012) for a full exploration of the most common causal frameworks (counterfactual, regularity, configurational and generative).
